# Deficits of Affect Mentalization in Patients with Drug Addiction: Theoretical and Clinical Aspects

**DOI:** 10.1155/2013/250751

**Published:** 2013-11-11

**Authors:** Svetoslav Savov, Nikola Atanassov

**Affiliations:** Department of Cognitive Science and Psychology, New Bulgarian University, 21 Montevideo Boulevard, 1618 Sofia, Bulgaria

## Abstract

Traditionally treated with wariness, drug addictions have provoked a serious interest in psychodynamically oriented clinicians in recent decades. This paper discusses the development of contemporary psychodynamic conceptualizations of addictions, focusing specifically on mentalization-based theories. The concept of mentalization refers to a complex form of self-regulation which includes attribution of psychological meaning to one's own behavior and affective states, as well as those of the others. We hypothesize that drug-addicted patients have severe impairments in mentalizing, associated with developmental deficits, characteristic for the borderline personality disorder and psychosomatic conditions. Psychodynamic models of mentalization and their corresponding research operationalizations are reviewed, and implications for a contemporary understanding of drug addictions and psychotherapy are drawn. The authors propose that mentalization-oriented theories provide an adequate conceptualization, which is open to empirical testing and has clear and pragmatic guidelines for treatment.

## 1. Introduction

We know that the onset of drug addictions is determined by a complex combination of constitutional (biological), social, and psychological factors. Physiological components play a crucial role in the maintenance of psychological anxiety, associated with physical abstinence, but they are not the one and only etiological factor behind this disorder and they can not entirely explain the motivation for subsequent relapses [1]. It is clear that every psychoactive drug induces a specific state of intoxication, but individual psychopathology largely defines the subject's reaction towards the pharmacological effects [2].

Bearing in mind these assumptions, we review a series of developments in the understanding of substance abuse starting with classical psychodynamic approaches based on drive/conflict models. Then, we present comprehensive theories of affect regulation which we see as a starting point for the transition to modern mentalization-based conceptualizations. We describe the shift from the initial emphasis on instinctual gratification to the investigation of ego development and pathology. We show how contemporary psychodynamic clinicians and researchers can increasingly rely on mentalization based theories to explain personality pathology. We outline some of the implications of this shift for evidence-based practice.

There is clear evidence supporting neurobiological and neurocognitive adaptations to specific drug exposure, the natural history of addiction involving “spontaneous recovery” with minimal intervention, consideration of now copious evidence from other therapeutic interventions such as motivational interviewing that appear to also emphasize the interpersonal environment in treatment. However, these issues are beyond the scope of the current article. Our main aim is to examine the evolution of psychodynamic approaches to addictions which we see as parallel to the general development in psychodynamic clinical theories.

## 2. Classical Psychodynamic Approaches to Addictions

Many psychoanalytic pioneers were interested in the problem of substance abuse and addictions. Abraham [3] tried to conceptualize this form of pathology from the point of view of the libidinal theory, that is, as a symptom of regress to oral fixations and striving for “orgasmic” experiences. Radó [4] was the first to point out that not the toxic agent itself, but the impulse to use it, defines addictions. Fenichel [5] underlined the deep-seated depression and anxiety in addicts.

 In general these authors understood substance abuse from the point of view of euphoric-pleasurable experiences and believed that the symptom has a “hidden” meaning (e.g., symbolizing an orally gratifying object). Glover [6], however, made an important breakthrough with his hypothesis that the psychoactive substance could be used “progressively” not only for regressive satisfaction, but also for protecting the subject from primitive (destructive and self-destructive) impulses or even psychosis. These ideas would later serve as a basis for Khantzian's work on the self-medication hypothesis [7].

## 3. Affect Regulation

The next generation of psychodynamic models comes largely from the ego psychological tradition. The main difference between ego-psychological theories and classical-drive/conflict models is that ego psychologists shift the focus from the symptom to the personality deficits of addicts and their incapability for coping with traumatizing anxiety. Drug use is related to specific ego pathology manifested in quick shifts from depressive states to intensive arousal in conflictual relations with important others [8]. Addicts react to situations of crisis with affect regression (totalization of feelings), which is dealt with by splitting unacceptable parts of internal or external reality and denying their existence. However, only when these series of operations are pharmacologically reinforced, a sense of mastery and raised self-esteem can be restored [9].

 This generation of psychodynamically oriented clinicians concentrates on the clinical reality that patients with drug addiction complain of being either overwhelmed by intolerably painful affects or cut off from their emotions. Referring to these characteristics of the affective life of addicts, Wieder and Kaplan [2] define drugs as “prosthesis” helping patients to regulate their impaired affective life.

The problem with drug addicts is that the primitive defense mechanisms they employ do not efficiently protect them from excessive anxious and depressive states. Consequently, addicts present not only interpersonal difficulties, affect storms, and impulsive behavior, typical for patients with borderline personality disorder, but their whole emotional life is in a way much more easily “somatized” and driven around bodily sensations. Thus, impaired affect regulation comes into focus as a central diagnostic feature of this disorder. This term denotes a qualitative transformation of affective states with modification of their intensity and/or duration [10]. It encompasses an interaction between neuro-physiological, motor-expressive, and cognitive-experiential areas. Constant defensive blocking of affectivity leads to a state in which emotions are treated as physiological attacks setting vicious circles such as “I am afraid to be afraid.”

 We can safely generalize that the contemporary psychodynamic approach to drug addictions abandoned altogether early conceptualization of pleasure seeking and symbolic importance of the drug. Instead, leading authors like Khantzian [7] see the motivation behind substance use as an attempt for “self-medication.” He observes that patients addicted to opiates rely on the antiaggressive effects of the substance which block disorganizing and threatening affective states of anger, pain, shame, and loneliness. Khantzian convincingly shows that regressive aspects of the psychopharmacological effect have been excessively stressed, leading to a neglect of its “progressive” functions, that is, blockage of primitive psychic states and reinforcement of ego defenses—drug addicts do not just search for an “escape” or “euphoria.” They actually need a shield that protects them from excess in anxiety.

 We are naturally interested in the developmental origins of these affective disorders. Krystal [11] points that only when the small child is protected from exposure to continuous trauma in early relations, it can develop affect tolerance during latency and adolescence. He makes it clear that primary self-regulation deficits in drug addicts encompass a tendency for affective regress, deficient capability for using anxiety as a signal, and impaired tolerance for painful emotions, especially the primitive affect of undifferentiated anxiety and depression.

 These deficiencies showing failure in the desomatization, verbalization, and symbolization of affective experiences are quite often observable in psychosomatic conditions characterized by the state of “alexithymia” (from Latin, “no words for emotion”) [12]. Addicted patients often share some of the following basic components of alexithymic functioning: (1) difficulty in identification and differentiation of feelings and bodily senses in a state of emotional arousal, (2) difficulty in describing feelings, (3) limited imagination and poor fantasy life, and (4) cognitive style focused on external reality [13].

 Without having a good enough understanding of their mental states, these patients cannot modulate emotions and instead show tendencies for direct discharge of anxiety in behavior or somatizations. Interpersonal disappointments easily trigger rapid changes in mood, which an individual with certain predispositions would try to regulate by pharmacological means. Interestingly, alexithymia—a concept highly applicable to addictions, is also one of the roots of contemporary mentalization-based theories.

## 4. What Is Mentalization?

 The concept of mentalization was introduced by Pierre Marty in the 70s as an extension of the research into psychosomatic phenomena [14]. Classically it refers to the quality and quantity of psychic representations, their verbalization, and connections with affectivity. From another point of view that of modern developmental psychopathology Fonagy [15, p. 4] defines mentalization as “… a form of mostly preconscious imaginative mental activity, namely, interpreting human behavior in terms of intentional mental states (i.e., needs, desires, feelings, beliefs, goals, purposes, and reasons).” This conceptualization integrates the notion of “theory of mind” in cognitive developmental models with attachment theory [16]. It is based on the following three main assumptions [17]: (1) the feeling of the self as agent is rooted in the experience of being attributed psychic states by a significant other, (2) this capability is a function of the interaction between the caring figures through a process of mirroring, (3) its development can be impaired by traumatic experiences.

 Attachment is seen as the main factor in the development of mentalization and the formation of internal representations of affective states. Secure attachment is a precondition for good enough affect regulation and guides the transition from coregulation in the mother-infant couple towards self-regulation of the child [10]. The child internalizes mother's empathic expression and this type of “intersubjectivity” is a milestone in the relation between attachment and affective self-regulation. Using language, children can name their feelings, receive verbal and emotional feedback about their adequacy, and thus become supported in the effort to think about themselves and others [13].

 Bateman and Fonagy [18] understand borderline personality disorder as a result of inadequate mirroring in early development. According to them, patients with borderline personality disorder have difficulty in mentalizing mainly in interpersonal and intimate situations, when they are most vulnerable to excesses in anxiety. Deficits in mentalization prevent them from having a good enough “buffer” from affects and trigger “fight or flight” mechanisms. These observations are relevant for the conceptualization of addictions, having in mind the high percent of comorbidity with borderline personality disorder [19].

Allen et al. [20] describe a two-way interaction between substance abuse and mentalizing. Intoxication impairs mentalization of own emotional states as well as those of the attachment figures. Deficits in mentalization on the other hand contribute to an inclination for substance abuse under emotional stress caused by interpersonal conflicts with attachment figures.

 Contrasting, but also complementing Fonagy's model, Bouchard and Lecours [21] present a theory of mentalization focused on the development of thinking through binding of instinctual pressure in representative networks. This theory has roots in the psychosomatic research done by Marty [22], Krystal's theory of emotions [11, 23], and Piaget's conceptualization of the child's intellectual development from sensor-motor activity towards formal verbal thought [24]. Bouchard and Lecours describe a complex process of psychic working through which prevents direct discharge into actions or somatizations. Affects are here conceptualized as positively or negatively valanced psychological phenomena with a somato-motor tendency for action. This tendency is “desomatized” by a complex process of psychic working through. The authors assume that representative deficits lead to an excess of excitation, which has not been transformed into a psychic conflict, that is, anxiety has not been mentalized. Forms of impulsivity, addicted behavior, and somatization are interpreted from this point of view as an expression of accumulated drive impulses with no attributed psychological meaning.

 Bouchard and Lecours [21] describe mentalization as a continuous process, functioning as “immune system” of the psyche, because it modifies external and internal pressures. Normally, mentalizing contributes to the coherent and meaningful experience of one's own psychic states. Instead of acquiring this tolerable distance from direct affective pressure, drug addicts often suffer from severe anxiety and depression. These conditions are triggered by the deep conviction that the individual is helpless in regulating not only external reality but also his or her emotional states. Substance abuse is the coping mechanism which replaces mental processing of helplessness, apathy and emptiness, and thus brings back temporary control.

## 5. Empirical Research on Mentalization in Addicted Patients

A considerable body of research on disorders of affect regulation has been accumulated. Bateman & Fonagy [25], for example, point that patients with alexithymia usually grow in families characterized by high levels of negative affect, difficulties in representation, and impaired recognition of emotional expressions, as well as scant discussion of emotions in the parent-child relationship.

 Haviland et al. [26] present more specific data about the relationship between alexithymia and substance use disorders. In a sample of 204 patients (72 treated for alcoholism, 79 for drug addiction, and 53 for double addiction) 41.7% were diagnosed as alexithymic, the percentage of alexithymia being significantly higher among women (50%) than the corresponding figure among men (35.8%). High levels of alexithymia in substance use disorders contrast low levels (9% and 12%) among male adults and students in norm, and 8% and 12% among women, respectively.

There is also a growing body of research supporting the foundations of mentalization theory in various clinical groups and ages. High mentalizing functioning is shown to protect the child from early traumatic influence [25]. MacBeth et al. [27] find data supporting the association between mentalization and social functioning in patients with psychosis. Taubner et al. [28] show that depressed patients tend to have lower reflective functioning scores concerning issues of loss compared to healthy controls. The cited research fails to show the relation between reflective functioning and symptomatology in depressed or psychotic patients. However, Lecours and Bouchard [29] present data that the severity of symptoms in the borderline personality disorder is associated with low level of mentalization in two distinct areas depressive and aggressive affects. Furthermore, traumatized individuals are likely to meet the DSM-IV criteria for borderline personality disorder only if they have deficiencies in mentalizing [30]. It is clear that the complex links between severity of symptoms and quality of mentalization should be further explored.

From the point of view of the relation between mentalization and stress, it is becoming clear that relative calmness supports mentalization, while excessive anxiety contradicts it [15]. An evident parallel exists with addictions, where an inability to contain affects, which is a basic component of mentalization, could be used as a key psychodiagnostic characteristic.

Looking at this promising line of investigations, it is surprising that there is hardly any published research on drug addicted patients' mentalizing functioning. Levy and Truman [31], however, report data showing that cocaine-using mothers' maternal mentalization mediates associations between maternal substance abuse and children's psychosocial development. Improvement in mentalization in response to a mentalization-based parenting intervention was associated with improvement in maternal caregiving behavior and increased regulation in children between 24 and 36 months of age. Ostler et al. [32] examine children exposed to parental methamphetamine abuse and find that those with higher mentalization were less prone to underreport symptoms, had fewer mental health problems, and were rated by their caregivers as more socially competent. 

The apparent scarcity of empirical research on mentalization in addicted patients stands in contrast to the fact that the presented models have already been operationalized successfully. The most widely used instrument for mentalization assessment is the Scale for Reflective Functioning in the Adult Attachment Interview (AAI) [33]. Low results on that scale are registered if the subject demonstrates poor access to the motivational origins of behavior.

The Grille de l'Élaboration Verbale des Affects (GEVA) [34] offers an alternative empirical approach. It measures verbal affect working through by segmenting and coding affective units in narratives. It consists of two orthogonal dimensions depending on the level of mentalization: four channels of affect expression (somatic and motor activity, imagery and labeling verbalization) and five levels of affect tolerance and abstraction (disruptive impulsion, modulated impulsion, externalization, appropriation, and meaning association). These are a total of 20 possible forms (4 channels × 5 levels) of affective expression. They are used to calculate an aggregate score for the quality of affective mentalization.

## 6. Implications for Treatment

There is a common agreement that the treatment of drug addictions has to offer an encompassing range of services—medical, social, and psychological. The place of psychotherapeutic work is without doubt central because it is directed at changing the psychic functioning of the patient. It is assumed that this happens through a gradual process which deepens the understanding of the place of addiction in one's own life.

It is well known that early attempts at psychotherapeutic work with addicted patients offered no base for optimism. We are familiar with these individuals' proneness to fall into relapse and drop out of therapy, as well as low tolerance for psychic pain combined with tendency for self-destructive behavior. Contemporary psychodynamic forms of treatment of personality disorders, however, show a promising approach, focused on the current mental states of the patient. Aiming to create a network of representations of these internal states, mentalization-based treatment (MBT) [25] follows a different path from the traditional psychoanalytic technique. It does not aim at interpreting “deep” unconscious fantasies but works instead with easily accessible content. The therapist's goal is to restore mentalizing by creating representational coherence and integration of mental states. The approach that Bateman and Fonagy offer is well structured and focused on enhancing the working alliance and paying attention to the immediate interaction between the patient and the therapist. The therapist is flexible to the specifics of the pathology (impulsiveness, interpersonal difficulties, self-destructive behavior, etc.) by being relatively active and encouraging a strong relation of attachment which makes it possible for both participants to explore and overcome current failures in mentalizing.

MBT explores the relationship between affect and belief and has some technical features similar to cognitive behaviour therapy, which explores the relationship between maladaptive schema and dysfunctional cognition or problematic feeling and has been widely used to treat drug addiction. Of course, to the extent of focusing on the “here and now” patient-therapist relationship, and of placing an emphasis on transference (albeit not interpreting it actively), MBT is still psychodynamic psychotherapy.

This approach has already been extensively studied in several randomized control trials with patients with personality disorders. Bateman and Fonagy [18], for example, report an 8-year followup of 41 patients with borderline personality disorder in partial hospitalization. MBT is associated with significant improvement in all measured areas (depressive symptoms, suicidal and self-destructive behavior, number and duration of hospitalizations, social and interpersonal functioning, and use of psychopharmacological medication). The improvement is sustained 5 years after the end of therapy, while patients in treatment as usual show limited change or even deteriorate during the same period.

This data should be considered having in mind that MBT requires relatively little additional training on top of general mental health training, and has been implemented in research studies by community mental health professionals, primarily nurses, with limited training given modest levels of supervision. This is an important aspect from the point of view of delivering cost-effective interventions. However, studies of the efficacy of this approach with drug addicted patients still have to be carried out.

## 7. Discussion

Disorders of affect mentalization in drug addicts find different explanations in the light of the “conflict-deficit” dilemma. Authors like Khantzian stay closer to the mentalization models and see the lack of self-regulatory functions as an ego deficit, that is, a function that was never established, while, according to Krystal, self-caring is “forbidden” by archaic parental figures. In that sense, Krystal's position is analogous to the classic psychoanalytic perspective, explaining addictions as a result of unconscious conflicts.

Following Fonagy's work and staying more closely with Khantzian we understand low abilities for mentalization in addicted patients as a personality deficit related to early environmental failures. From this point of view mentalization-based theories stress predominantly the relational aspects in the functioning of the patients against the intrapsychic dimension of the traditional psychodynamic theories. Usually these individuals share a history of interaction with a caregiving figure, which had not provided good enough mirroring or it was discontinued due to a trauma. This focus on the direct interpersonal environment, typical for Fonagy's theorizing, however, has been criticized for an underestimation of complex object relations which modulate affect activation [35]. Indeed, de-emphasis of the unconscious is a central feature in Fonagy's theory. At the same time, as Diamond and Kernberg also point out, Fonagy and his colleagues show significant empirical evidence that mentalization is established in the context of secure attachment and is blocked in insecure attachment and/or severe trauma.

Choi-Kain and Gunderson [36] add several critiques to the mentalization theory based on the idea that this concept is too broad and multidimensional. However, its core measure—the Reflective Functioning Scale, yields only a single score and it is time-consuming to administer. The authors propose that research should focus on more limited concepts for which self-report measures have been developed (i.e., theory of mind, mindfulness, empathy, etc.). Another limitation, according to them, in the Fonagy model is emphasizing process over content.

Other authors (i.e., [37]) have been trying to answer the question whether the concept of mentalizing is just a new word for well-known phenomena. It becomes clear, however, that it offers a pragmatic integration of clinical observations, developmental psychopathology, and attachment experiences. It is also a useful explanatory framework, connecting affect regulation and clinical symptoms. Last but not least, mentalization combines psychodynamic hermeneutics with evidence-based practice and is open to advances in contemporary neurocognitive theories.

 Strength in Fonagy's mentalization model and the classically oriented model of Bouchard and Lecours [21] can be regarded as genuinely integrative theories uniting the intersubjective-relational approach with the drive/conflict perspective. An important point of distinction, however, is that Fonagy's theory underlines the interaction with a real object attributing meanings to the child's affective states, while Bouchard and Lecours focus primarily on the role of representative mechanisms in verbal affect mentalization. So, although the two perspectives are not completely contradictory, fundamental in Bouchard and Lecours' theory are the organizing structures of unconscious subjective experience.

 Looking at the empirical evidence, we can see that these two approaches are distinct but also overlapping [38]. High levels of reflective functioning or elaboration of affect contribute to a basic feature of mentalization the capacity to treat behavior as driven by internal mental events and not just external stimuli.

## 8. Conclusion

Affect processing is a basic component of mentalization. Deficits in capabilities for affect representation and modulation manifested in drug-addicted patients point to similar psychogenetic origins of mentalization disorders with psychosomatic conditions. Better clinical understanding of this form of pathology is a key ingredient in the assessment and clinical work with drug-addicted patients. Based on a coherent understanding of personality organization, mentalization models offer promising potential for psychodiagnostic accuracy and therapeutic efficacy. Their corresponding operationalized instruments can be used as valuable tools for assessment of change in psychic functioning resulting from treatment. Further work is recquired on distinguishing between different subgroups in the addicted population (i.e., a comparison of specific impairments of the reflective function and pathways to addiction in opiate and stimulant users).
